# One-year clinical outcomes after fixation of condylar head fractures using a reduced-head PEO-modified WE43 biodegradable magnesium screw

**DOI:** 10.3389/froh.2026.1902807

**Published:** 2026-07-28

**Authors:** Henry Leonhardt, Michaela Bučkova, Nadja Kalies, Carsten Rendenbach, Jan Bernard Matschke, Adrian Franke

**Affiliations:** 1 Department of Oral and Maxillofacial Surgery, Medical Faculty and University Hospital Carl Gustav Carus, Technische Universität Dresden, Dresden, Germany; 2 Department of Oral and Maxillofacial Surgery, Charité – Universitätsmedizin Berlin, Freie Universität Berlin and Humboldt-Universität zu Berlin, Berlin, Germany

**Keywords:** biodegradable implants, bone remodeling, magnesium, mandibular condyle, fracture osteosynthesis, three-dimensional imaging

## Abstract

**Introduction:**

Surgical management of condylar head fractures (CHF) remains challenging, and the optimal fixation material is still debated. Biodegradable magnesium alloys may serve as an alternative to titanium, eliminating the need for implant removal. This study evaluated clinical and three-dimensional (3D) radiographic outcomes of a reduced-head, cannulated WE43 magnesium-alloy screw with a plasma electrolytic oxidation surface (HBS-Mg).

**Methods:**

In this retrospective single-arm, single-centre cohort study (STROBE-compliant), 20 patients with 22 CHFs were treated using HBS-Mg. Clinical outcomes (mouth opening, mandibular mobility, occlusion) and 3D radiographic parameters (condylar volume, surface area, and surface congruence assessed by root mean square [RMS] deviation) were analysed over a 1-year follow-up.

**Results:**

Maximum mouth opening improved significantly from 23.6 ± 6.9 mm (<50 days) to 45.1 ± 5.9 mm (>360 days; *p* < 0.0001), with stable occlusion achieved after 100 days. No permanent facial nerve palsy or implant-related complications occurred. Condylar volume and surface area decreased significantly (−24.0 ± 14.2%, *p* = 0.0051; −24.6 ± 11.9%, *p* < 0.0001). Surface deviations ranged from 1.4 to 1.7 mm, mainly during the first six months.

**Conclusion:**

HBS-Mg fixation yielded favourable clinical outcomes and stable functional recovery despite significant 3D condylar remodelling during the first postoperative year. The observed remodelling indicates that the use of a reduced-head, PEO-coated magnesium screw did not prevent postoperative condylar changes, although these changes were not associated with functional impairment within the study period.

## Highlights

- Magnesium screws enabled stable CHF fixation without complications
- Mouth opening improved significantly over 12 months
- No permanent nerve injury or implant-related adverse events
- Significant condylar remodelling (∼24% volume and surface area reduction)
- 3D changes showed no clinically relevant functional impairment

## Introduction

Mandibular fractures account for a substantial portion of maxillofacial injuries (1–3), with the condylar head being among the most frequently affected sites (4–6). As a vital component of the temporomandibular joint, which connects the mandible to the skull, this region holds particular clinical importance (7). Damage to the condylar head may cause functional impairments such as limited mandibular movement and occlusal disturbances, affecting daily activities like eating.

Management of condylar head fractures (CHF) includes conservative approaches as well as closed or open reduction with internal fixation (ORIF). Closed treatment usually requires mandibular immobilisation, most often by intermaxillary wiring for approximately 10 days, followed by functional elastic guidance. In contrast, internal fixation may involve only brief intraoperative or short-term postoperative immobilisation. This shorter period of temporomandibular joint immobilisation can help healthcare professionals feel confident in the efficacy of surgical options, as it substantially reduces overall treatment duration and facilitates early functional rehabilitation (8).

Surgical management of condylar head fractures is particularly indicated when there is a loss of vertical height of the ascending mandibular ramus, which guides clinicians in decision-making. This scenario is typically encountered in type p fractures according to the current AOCMF classification system (9, 10).

Over time, a wide variety of fixation methods have been proposed for this anatomically demanding region. These include mini- and microplate systems (11–13), mono- and bicortical titanium screws (14–16), small-fragment screws (17–20), biodegradable polylactide pins (21–24), and magnesium-based screws (25–30). Given the large number of available osteosynthesis techniques, direct comparison between them is challenging, highlighting the need for research strategies that integrate imaging, clinical and functional assessments, instrumental measurements, and experimental data (31–34).

Earlier studies using titanium cannulated screws reported some instances in which screws “migrated” within the condylar head. A certain degree of remodelling of the condylar head led to penetration of the articular surface into the joint space, necessitating removal of the fixation material (35). These drawbacks have already been addressed by utilising cannulated screws comprised of a biodegradable magnesium alloy (29). More importantly, the technique and material used display excellent results both clinically and radiographically (36) in long-term follow-ups. However, there were statistically significant changes in the volume, surface area, and three-dimensional (3D) morphology of the healing condylar head (30).

Among biodegradable magnesium implants, the WE43 alloy combined with plasma electrolytic oxidation (PEO) surface modification has attracted particular interest because it provides favourable mechanical properties and improves corrosion behaviour. The PEO coating forms a stable, ceramic-like surface layer that slows the initial degradation rate, enhances corrosion resistance, and promotes more predictable implant resorption while maintaining mechanical integrity during the early phases of fracture healing. Experimental and translational studies have further demonstrated favourable biocompatibility, osseointegration, and bone healing characteristics of PEO-coated WE43 implants, supporting their application in load-bearing craniofacial osteosynthesis (37–40). These characteristics suggested that combining a reduced implant volume with a PEO-modified WE43 alloy might reduce postoperative condylar remodelling while preserving adequate fixation stability.

The present study aimed to evaluate the clinical and radiographic outcomes of a slender, headless, cannulated magnesium-alloy screw used to treat CHF. The primary hypothesis was that reducing the amount of biodegradable material within the condylar head, together with the PEO-modified implant, would reduce postoperative condylar remodelling, as assessed by changes in condylar volume, surface area, and 3D morphology, while maintaining satisfactory functional rehabilitation. This hypothesis was tested using longitudinal clinical assessment and quantitative 3D radiographic analysis over a 1-year follow-up period.

## Materials

### Study design

In accordance with the STROBE (Strengthening the Reporting of Observational Studies in Epidemiology) guidelines (41), this single-centre retrospective cohort study (single-arm, without a control group) included patients with condylar head fractures treated at the Department of Oral and Maxillofacial Surgery, Carl Gustav Carus University Hospital Dresden, between July 2023 and December 2024.

Eligible patients met predefined inclusion criteria: diagnosis of a CHF with an indication for ORIF according to institutional treatment protocols (primarily fractures associated with loss of vertical ramus height, typically corresponding to AOCMF type p fractures, although the final indication for surgery was made individually based on fracture morphology and clinical assessment), surgical treatment using at least one magnesium-based cannulated compression screw (HBS-Mg), provision of written informed consent (either personally or via a legal representative), and completion of a minimum 6-month postoperative follow-up with at least two radiographic assessments. Patients were excluded if no HBS-Mg was used for osteosynthesis, the follow-up was terminated before the 6-month follow-up, there were fewer than two radiographic assessments, or if they declined participation.

The study was conducted in accordance with the Declaration of Helsinki. Ethical approval was granted by the Ethics and Institutional Review Board of the University Hospital Carl Gustav Carus, Technical University of Dresden (IRB00001473; IORG0001076; internal ethics committee ID: BO-EK-1012022). Informed consent was obtained from all participants and/or their legal guardians prior to intervention.

### Implant

**Figure 1:**
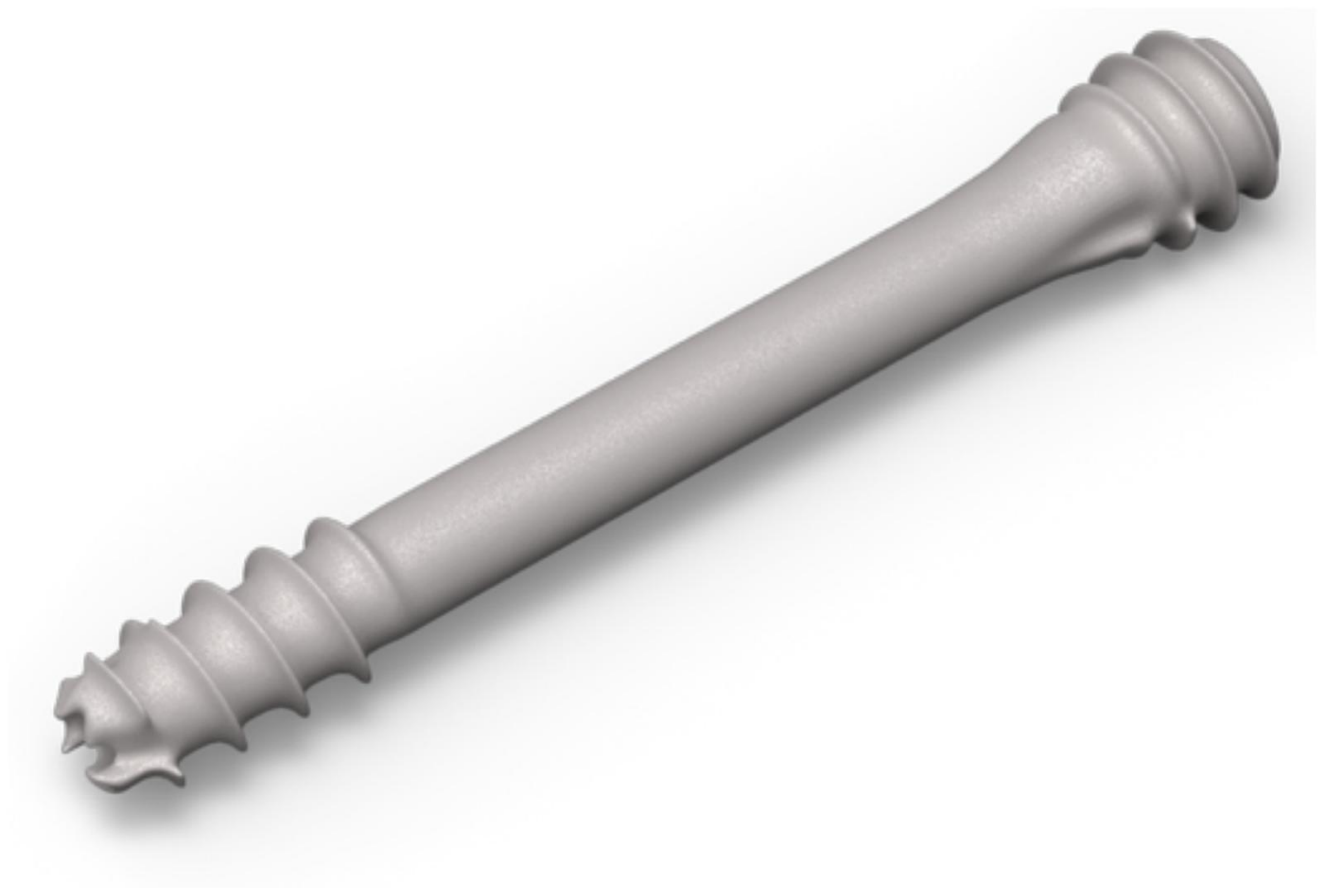
Detailed depiction of the HBS 2 resorb Mg. Source: KLS Martin SE & Co. KG. Reproduced with permission from KLS Martin SE & Co. KG, KLS Martin Platz 1, 78532 Tuttlingen, Germany. © Copyright KLS Martin SE.

The implant used for all participants in the study is the HBS 2 Resorb Mg mini (HBS 2 Resorb Mg mini, Ø 2.5 mm, short thread, article number: 53-800-1x-04; KLS Martin SE & Co. KG, Tuttlingen, Germany), composed of a WE43 magnesium alloy with PEO-modified surface (Figure 1). The material's efficacy and appropriateness have already been characterised in several preclinical and translational studies (37–40).

**Figure 2:**
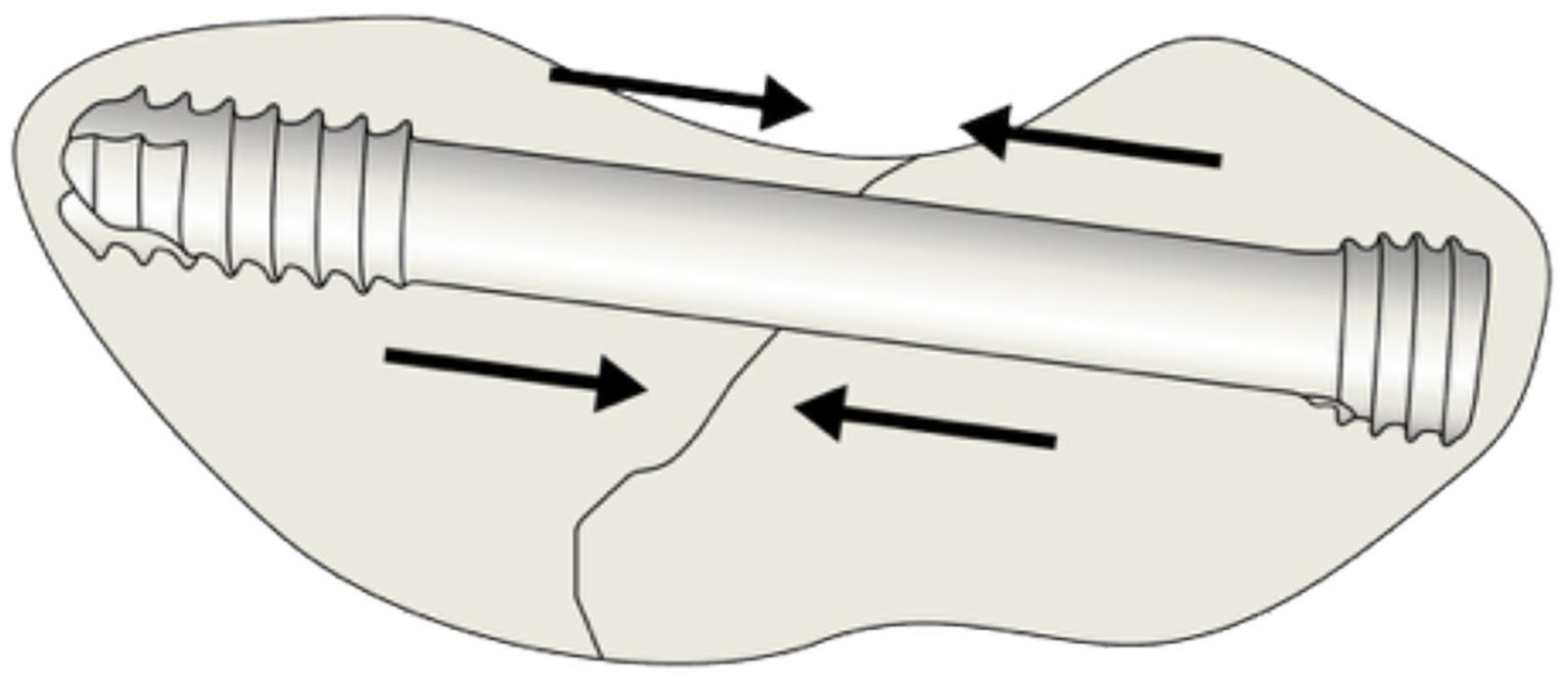
Schematic depiction of the principle of compression osteosynthesis using a headless cannulated bone screw. Source: KLS Martin SE & Co. KG. Reproduced with permission from KLS Martin SE & Co. KG, KLS Martin Platz 1, 78532 Tuttlingen, Germany. © Copyright KLS Martin SE.

Owing to its cannulated design, the screw enables envelope-type fixation, allowing accurate placement of the implant at the target site. Different thread pitches at the tip and head generate interfragmentary compression once each thread engages its respective bone fragment, thereby providing strong fixation stability and supporting fracture healing (Figure 2).

### Surgical procedure

**Figure 3:**
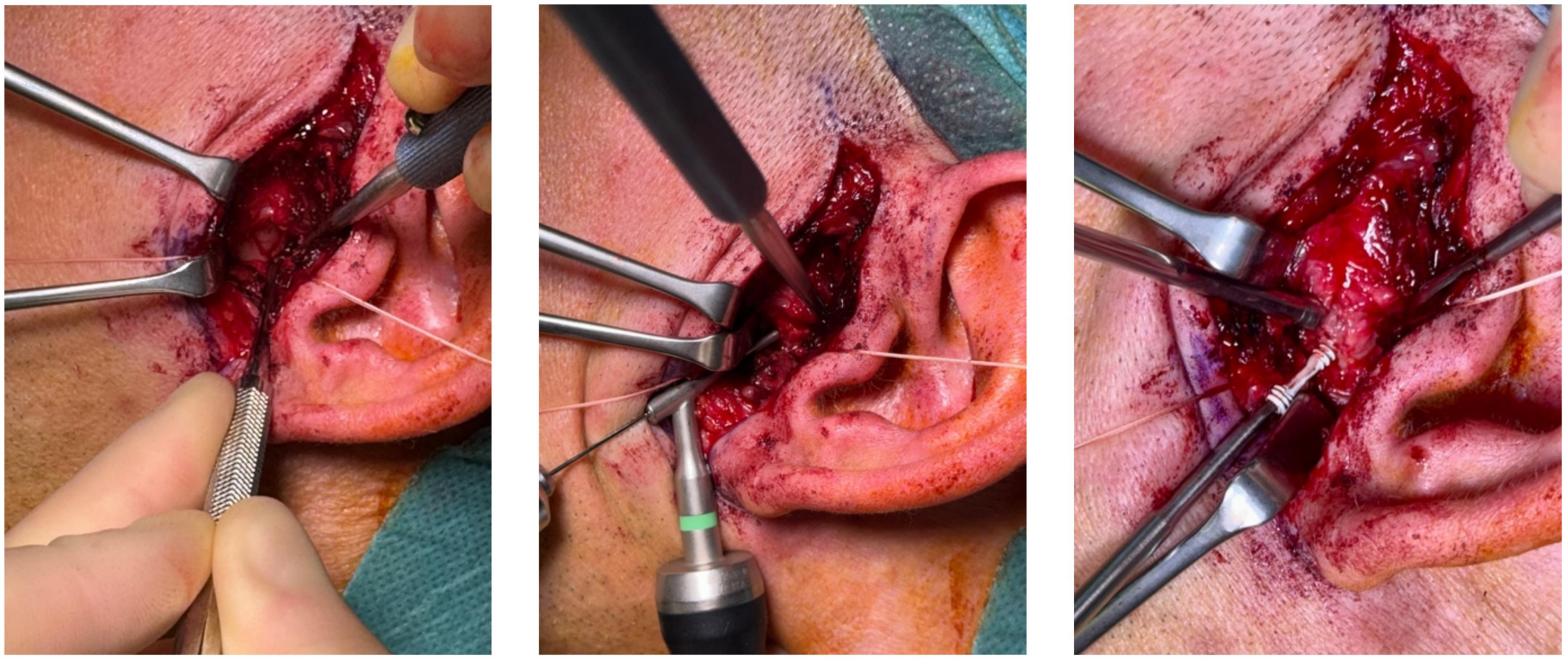
Photographs of several steps during the surgical procedure. Left: Preauricular approach on the left side. The joint capsule was opened, and the fracture was reduced. Middle: A trocar deflects the soft tissues to aid the temporary fragment fixation via a Kirschner wire. Right: the HBS-Mg is inserted over the Kirschner wire and completely sunk into the bone of the mandibular neck.

Implantation of the HBS-Mg follows the same principles as for conventional headless bone screws. A preauricular approach is used to expose the fracture site. After mobilisation of the anteromedially displaced condylar head fragment, the fracture is reduced and temporarily stabilised with one or two 0.9 mm Kirschner wires. A cannulated drill is then used to prepare the canal in the condylar neck. Particular care is taken not to advance the drill into the proximal condylar head fragment in order to maximise anchorage of the screw tip threads within that fragment. The HBS-Mg is subsequently advanced over the guide wire, and interfragmentary compression is generated at the end of insertion as the different thread pitches at the screw tip and head engage the surrounding bone. The implant is fully countersunk within the bone to avoid contact between the magnesium alloy and adjacent soft tissues (Figure 3). All procedures were carried out by at least one senior consultant in oral and maxillofacial traumatology to ensure patient safety. Postoperatively, elastic intermaxillary fixation was applied for two days to minimise the risk of joint effusion (28–30).

### Follow-up

Follow-up visits were organised into predefined time clusters: Cluster 1 (0–50 days), Cluster 2 (51–100 days), Cluster 3 (101–200 days), Cluster 4 (201–359 days), and Cluster 5 (≥360 days) (30, 35). Clinical outcome parameters included the number of missing teeth, maximum mouth opening, mandibular mobility, and occlusal status. Secondary outcomes covered patient-reported symptoms (e.g., pain, discomfort, impaired mastication, sensory disturbances, and unfavourable scarring) as well as objectively verifiable complications (such as facial nerve weakness and salivary fistula formation). Whenever possible, radiographic examinations were obtained during follow-up to assess osseous healing and to detect potential mechanical complications. Definitive points in time for 3D radiographic examination were during or shortly after ORIF (T1), six months after surgery (T2), and one year after surgery (T3).

Mandibular excursions and maximum interincisal opening were measured using a calliper and recorded in millimetres. To assess occlusion, a metallised polyester foil (Arti-Fol® 8 μm, Dr Jean Bausch GmbH & Co. KG, Cologne, Germany) was inserted between all opposing teeth. Occlusion was classified as adequate when the foil was retained between all antagonistic tooth pairs posterior to the canines. In contrast, it was deemed inadequate if the foil could be withdrawn without resistance.

### Radiological examination

The image acquisition, segmentation workflow, reference-plane definition, and three-dimensional analysis were performed using the same methodology as in our previously published magnesium- and titanium-screw studies to facilitate methodological consistency across investigations (30, 35): Intraoperative or immediate postoperative verification of fracture reduction was performed using cone-beam computed tomography (CBCT). In the operating room, imaging was obtained with the xCAT® ENT system (Xoran Technologies LLC, Ann Arbor, MI, USA). Postoperative 3D scans were acquired with the Planmeca Viso® G7 (Planmeca Oy, Asentajankatu 6, FI-00880 Helsinki, Finland) using a voxel size of 0.45 mm. Additional radiographic examinations were carried out during follow-up. DICOM datasets were exported and processed for further analysis.

All radiographic data were manually segmented without the use of algorithms or artificial intelligence, applying the Elements Contouring 4.5.0 workflow (Brainlab AG, Munich, Germany) to delineate temporomandibular joint structures from the DICOM files. The resulting segmented models were then imported into Artec Studio 19 Professional x64 (version 19.2.2.54) from Artec 3D (Artec 3D, 20 rue des Peupliers, L-2328, Luxembourg) for further processing. Individual segmented models were aligned in the ramus area, while the mandibular condyle was excluded from the matching procedure. The models were subsequently cropped along the A-line (42), which runs perpendicular to the posterior border of the mandibular ramus and passes through the most inferior point of the incisura semilunaris (30, 35).

**Figure 4:**
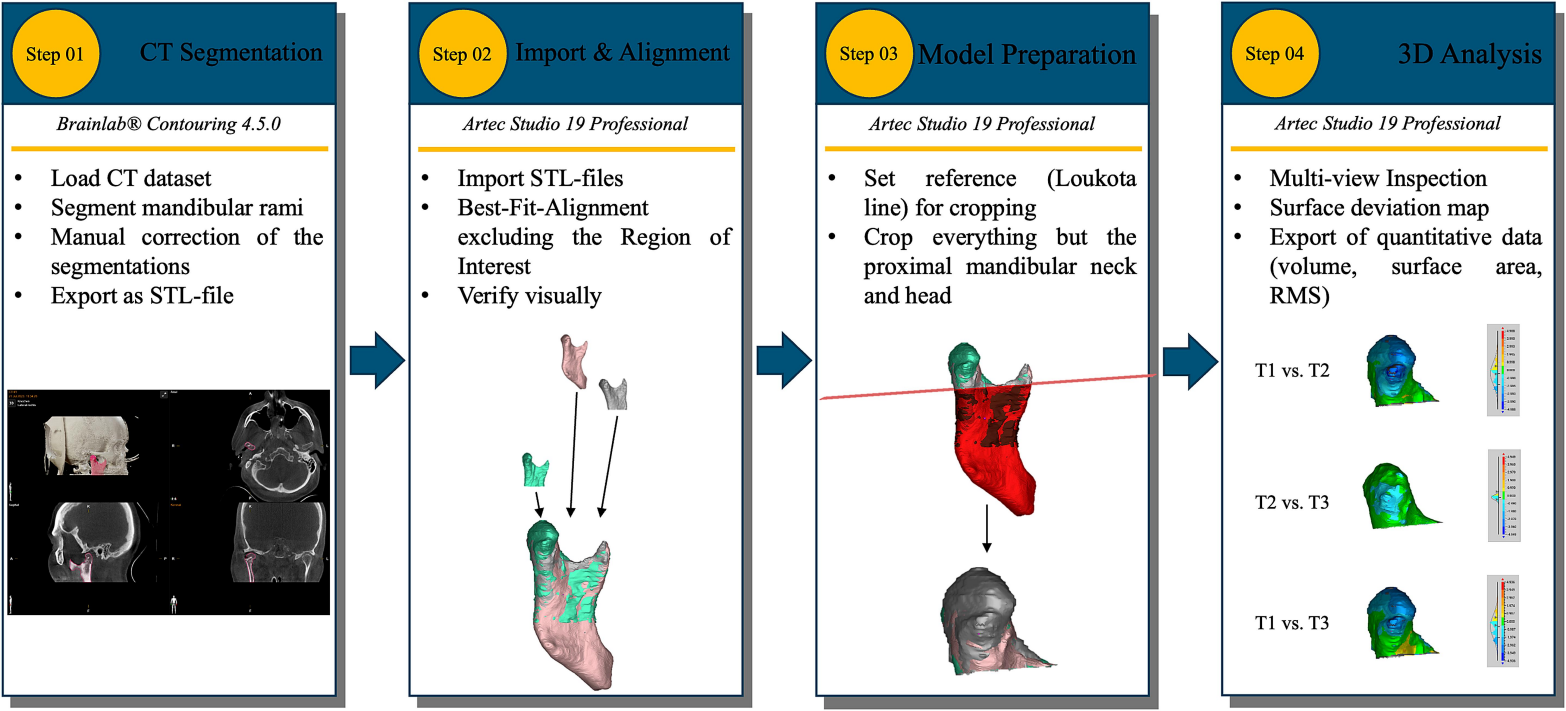
Schematic of the workflow to obtain the three-dimensional quantitative data.

**Figure 5:**
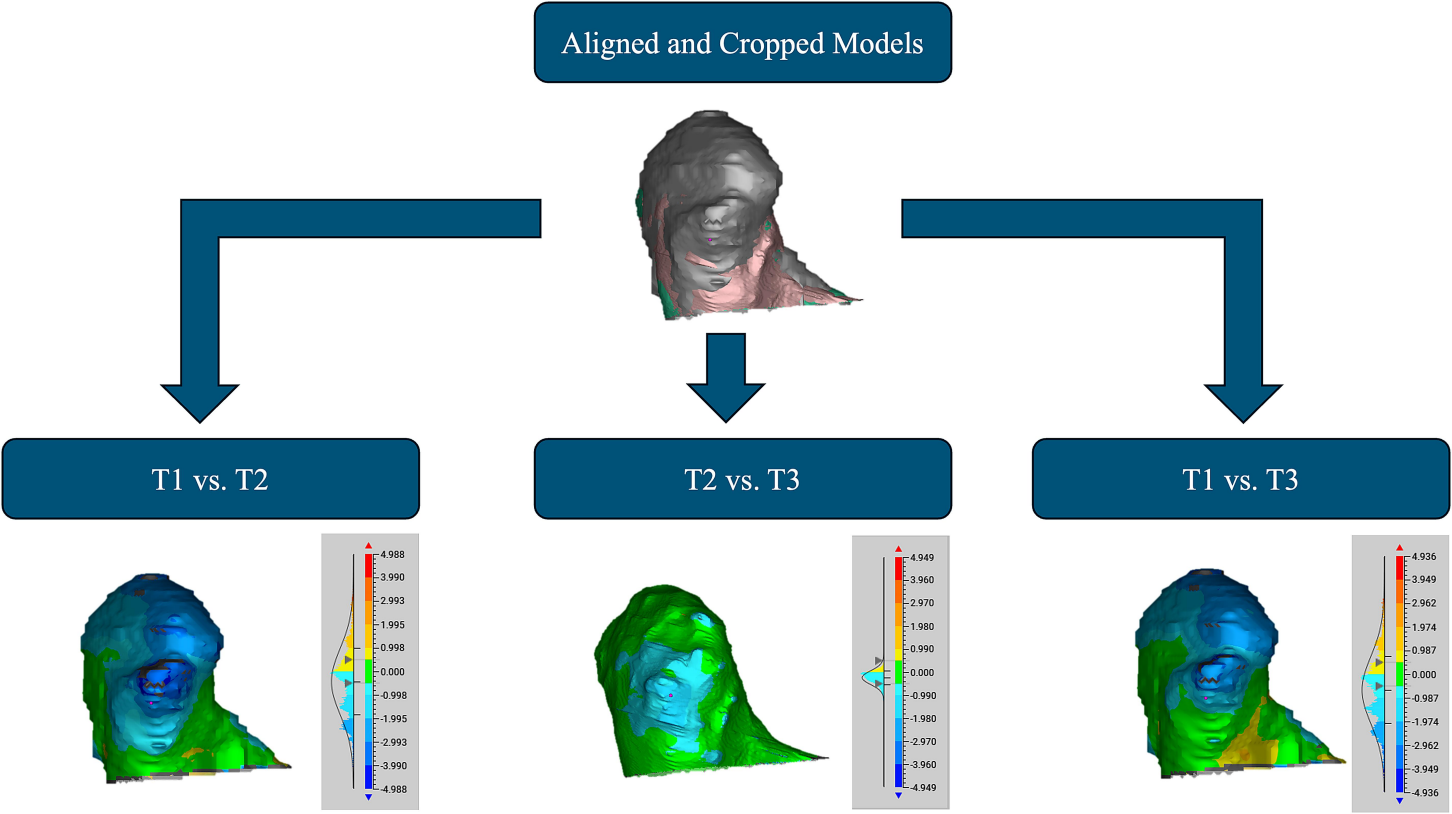
Representative three-dimensional surface comparison between postoperative time points. Colour-coded heatmaps illustrate local surface deviations relative to the reference model. Green indicates minimal surface change, warm colours (yellow–red) indicate increasing positive deviations, and cool colours (light blue–dark blue) indicate increasing negative deviations. The magnitude of deviation is expressed in millimetres according to the colour scale.

The resulting region of interest models comprised the complete condylar head, together with a small portion of the condylar neck. For each radiograph, the volumes and surface areas of the region of interest models were recorded. To quantify temporal changes, models from different time points were compared using the root mean square deviation (RMS). For this purpose, point clouds were generated on both segmented surfaces, and the distances between corresponding points were calculated. Lower RMS values indicated greater surface congruence, whereas higher values reflected increasing discrepancies between the models. Results were expressed in millimetres. To facilitate spatial interpretation, colour-coded heatmaps were generated for the 3D models (Figure 4). Warmer colours (yellow to red) indicate greater positive deviations from the reference model. In contrast, cooler colours (light blue to dark blue) represent negative deviations. Green denotes regions with minimal or no measurable surface change (Figure 5). Similarly, in addition to the RMS, the mean absolute distance (MAD) and the mean signed distance (MSD) between corresponding surface points were calculated.

### Statistical analysis

All patients meeting the inclusion criteria during the study period were included to avoid selection bias, making *a priori* sample size calculation unnecessary. Additionally, as this was a retrospective study based on existing clinical records, the sample size was fixed by the number of available eligible cases and could not be prospectively planned.

To minimise intra- and interobserver variability, repeated measurements of volume, surface area, and 3D parameters for congruence were obtained from a randomly selected patient in the database at two separate time points by two independent examiners. Reliability was assessed using the intraclass correlation coefficient (ICC) based on a two-way random-effects analysis of variance (ANOVA) model for single-rater type, with absolute agreement (43).

In addition to descriptive analyses, all data were evaluated using Prism 11 (version 11.0.0; GraphPad Software, San Diego, CA, USA). Continuous variables were summarised as means with standard deviations or as medians with interquartile ranges (IQRs), while categorical variables were reported as absolute numbers and percentages. Relative differences or changes were computed according to the following equation:

$$Relative\;Difference\;"value"\;=\;\left|\frac{\;("value"\;first\;exam.\;-\;"value"\;last\;exam.)}{("value"\;first\;exam.\;+\;"value"\;last\;exam.)\;/\;2}\right|\;\;x\;100\text{\%}$$

Data were displayed graphically when appropriate. Normality was assessed with the Shapiro–Wilk test. For univariate analyses, continuous variables were compared using ANOVA if they followed a normal distribution and the Kruskal–Wallis test if they did not. Ordinal and categorical variables were analysed with Fisher's exact test. Statistical significance was defined as *α* = 0.05. Correlations were examined using Spearman's rank correlation coefficient. To identify potential predictors, multivariable linear and logistic regression models were applied, with age and sex specified as the independent variables. Because of the limited sample size and the resulting small numbers within individual AOCMF fracture subtypes, no stratified analyses according to fracture classification were performed. Consequently, fracture subtype was not included as a covariate in the multivariable models, and analyses should be interpreted as exploratory. Where relevant, 95% confidence intervals were reported.

## Results

### Patients

A total of 20 patients were analysed, including 9 men (45.0%) and 11 women (55.0%), corresponding to a male-to-female ratio of 0.8:1. The mean age of the cohort was 55.7 ± 15.0 years (median 54.0, 95% CI 48.7–62.7), with ages ranging from 25 to 86 years. Male patients had a mean age of 54.3 ± 15.7 years (median 49.0, 95% CI 42.3–66.4), while female patients were on average 56.8 ± 15.0 years old (median 54.0, 95% CI 46.7–66.9); this difference did not reach statistical significance (unpaired t-test: difference between means −2.5 ± 6.9; t(18) = 0.3608, *p* = 0.7224). Throughout the results section, sex-stratified analyses were performed only as exploratory analyses due to the limited subgroup sizes (9 men and 11 women). Consequently, these comparisons were not powered to detect moderate between-sex differences and should be interpreted with caution.

The most frequent causes of admission were falls, accounting for 12 cases (60.0%), followed by traffic-related injuries in 7 patients (35.0%). One case (5.0%) was reported as a leisure-related accident.

### Fractures

In total, the 20 patients presented with a total of 22 condylar head fractures, including two patients (10.0%) with bilateral involvement. Eleven fractures (50%) occurred on the left side and eleven fractures on the right side. A detailed distribution according to the AOCMF classification is provided in Table 1 (10).

**Table 1:** Number of CHFs according to the AOCMF Level 3 classification.

	Level 3 classification	Amount (*n*)	Relative (%)
All patients	Hp0	4	18.2
	Hp1	13	59.1
	Hp2	4	18.2
	Hm0	1	4.5
	Total (*N*)	22	100.0

### Follow-up

Patient follow-up rates were 100.0% in Clusters 1-4 and 60.0% in Cluster 5. In the long-term follow-up group (Cluster 5), the interval between surgery and evaluation ranged from 360.0 to 437.0 days (0.99–1.20 years) and averaged 389.2 ± 27.0 days (95% CI 372.0–406.3), corresponding to 1.07 ± 0.07 years (95% CI 1.02–1.11).

### Clinical data

9 patients (45.0%) had no missing teeth, whereas 11 patients (55.0%) presented with missing teeth, resulting in a mean of 5.5 ± 7.4 missing teeth (median 3.5; 95% CI 2.0–8.9). Of these, seven patients (63.6%) had sufficient dental restorations, resulting in an adequate number of occluding pairs of teeth for clinical occlusal assessment. In the remaining four patients, the absence of adequate dental restoration limited conventional assessment of occlusal contacts.

Adequate occlusion was observed in 86.7% of patients in Cluster 1, 94.4% in Cluster 2, and 100.0% in all patients in subsequent follow-up intervals. Across the follow-up intervals, significant improvements were noted in maximum mouth opening and protrusion movements. There were statistically significant increased distances in the laterotrusive movements of the healthy and fractured sides. Except for the first follow-up, there were significant differences in laterotrusion between the healthy and fractured sides (Tables 2, 3).

**Table 2:** Clinical parameters mouth opening and protrusion, their development over time, and their statistical significance.

Clinical parameter	Median; Mean ± SD	95% CI of mean
Mouth opening		
Cluster 1	24.5; 23.6 ± 6.9	19.6 to 27.5
Cluster 2	30.0; 30.3 ± 6.1	27.4 to 33.1
Cluster 3	42.0; 40.7 ± 5.4	38.2 to 43.2
Cluster 4	42.0; 44.1 ± 6.1	41.2 to 47.1
Cluster 5	45.0; 45.1 ± 5.9	41.5 to 48.6
ANOVA	F = 60.847, df = 2.068, *p* < 0.0001^a^	
Protrusion		
Cluster 1	4.0; 4.0 ± 1.6	2.0 to 6.0
Cluster 2	4.0; 4.3 ± 1.8	3.4 to 5.2
Cluster 3	6.5; 6.5 ± 1.6	5.8 to 7.3
Cluster 4	6.0; 7.0 ± 2.0	6.0 to 7.3
Cluster 5	8.0; 7.8 ± 2.0	6.6 to 9.0
ANOVA	F = 23.692, df = 1.578, *p* < 0.0001^a^	

a Mixed-effects analysis.

**Table 3:** Overview of distances in mm for lateral mandibular excursions.

Time cluster	Laterotrusion with healthy condylar head		Laterotrusion with treated condylar head		Paired t-test *p*-value	Mean of differences ± SD; 95% CI
	Mean ± SD	95% CI	Mean ± SD	95% CI		
Cluster 1	6.4 ± 2.4	3.4 to 9.4	4.0 ± 1.2	2.5 to 5.5	0.0608	2.4 ± 2.1; −0.2 to 5.0
Cluster 2	7.6 ± 2.5	6.4 to 8.9	4.4 ± 2.3	3.3 to 5.6	< 0.0001	3.2 ± 1.8; 2.3 to 4.1
Cluster 3	9.9 ± 3.3	8.3 to 11.6	8.1 ± 3.5	6.4 to 9.8	0.0402	1.8 ± 3.5; 0.1 to 3.6
Cluster 4	9.8 ± 2.2	8.7 to 11.0	7.5 ± 2.7	6.1 to 8.8	0.0007	2.4 ± 2.3; 1.2 to 3.5
Cluster 5	11.1 ± 2.7	9.4 to 12.8	8.6 ± 3.6	6.3 to 10.8	0.0112	2.5 ± 2.8; 0.7 to 4.3
ANOVA	F = 7.947, df = 2.200 *p* = 0.0015^a^		F = 15.446, df = 1.693, *p* = 0.0002^a^			

a Mixed-effects analysis.

Transient facial nerve paresis occurred in none of the patients. A salivary fistula was detected in 1 patient (4.8%). In Clusters 3 and 4, 2 (9.5%) and 1 (4.8%) patients exhibited signs of craniomandibular dysfunction. Hypo- or dysaesthesia of the preauricular skin was reported by patients in all Clusters, with a maximum of 16 patients (76.2%) in Cluster 2 and a minimum of four patients (19.0%) in Cluster 5. One patient (4.8%) suffered from Frey's syndrome in Cluster 5. None of the patients reported subjective changes in overall well-being, and no soft-tissue emphysema or evidence of compromised bone stability was recorded. None of the patients required revision surgery or implant removal.

### Radiographic data

#### Intra- and interobserver bias

Repeated measurements demonstrated excellent (44) and good (43) agreement both between and within the two observers at the two independent time points, with intraclass correlation coefficients of 0.81 for volume, 0.82 for surface area, and 0.81 for the 3D parameters (RMS, MAD, and MSD).

#### Volume

There was a steady, statistically significant decrease across all patients over time (mixed-effects analysis: F (1.915, 31.60) = 6.402, *p* = 0.0051). Notably, statistically significant changes occurred between Clusters 1 and 2 (Tukey's multiple comparisons test: *p* = 0.0223) and Clusters 1 and 3 (Tukey's multiple comparisons test: *p* = 0.0460) (Table 4). No statistically significant changes in volume were detected comparing the male and female subgroups.

**Table 4:** Overview of the volume of the condylar head at the various examinations.

Volume of the condylar head (in mm^3^)		
	Mean ± SD	95% CI of mean
T1	2208.8 ± 659.8	1908.4 to 2509.1
T2	1895.9 ± 706.7	1574.2 to 2217.6
T3	1802.0 ± 625.2	1441.1 to 2163.0
Mixed-effects analysis	F = 6.402, df = 1.915, *p* = 0.0051	

Over the follow-up period, although the cohort showed a statistically significant overall decrease in condylar volume, individual patients exhibited both increases and decreases relative to baseline. Consequently, the reported relative changes represent the magnitude of individual remodelling rather than a uniform directional change across all patients. The mean absolute percentage of change in volume for all patients was 24.0 ± 14.2%, with a median of 20.7% and a 95% confidence interval of the mean ranging from 17.6–30.5% between the first and last recorded radiographs. There were no differences in relative changes in volume between male and female patients.

#### Surface area

The surface area of the condylar head showed a progressive decline over time (mixed-effects analysis: F (1.995, 32.92) = 19.469, *p* < 0.0001). *post-hoc* analysis using Tukey's test demonstrated significant differences between Clusters 1 and 2 (*p* = 0.0004), and Clusters 1 and 3 (*p* = 0.0002) (Table 5).

**Table 5:** Overview of the surface area of the condylar head at the various examinations.

Surface area of the condylar head (in mm^2^)		
	Mean ± SD	95% CI of mean
T1	1008.8 ± 192.0	921.4 to 1096.2
T2	833.7 ± 225.5	731.1 to 936.3
T3	772.5 ± 170.1	674.3 to 870.7
Mixed-effects analysis	F = 19.469, df = 1.995, *p* < 0.0001	

In male patients, there were no changes in surface area. However, in female patients, changes were observed across all time points (mixed-effects analysis: F (0.3899, 3.314) = 26.850, *p* = 0.0145).

As with volumetric measurements, surface area values fluctuated in both directions relative to baseline. The mean relative change in surface area was 24.6 ± 11.9%, with a median of 24.9% and a 95% confidence interval of 19.2–30.0%. There were no differences between male and female patients regarding the relative changes in surface area.

#### Three-dimensional congruence

The greatest RMS values were observed when comparing T1 and T2 radiographs; RMS values when comparing T2 and T3 radiographs were lower across all patients, including males and females (Table 6). Statistically significant lesser degree of change of the condylar head surface morphology was demonstrated between the comparison of T1 and T2, and the comparison between T2 and T3 (Wilcoxon matched-pairs signed rank test: median of differences = 1.064 mm; W = 97.00; *p* = 0.0009). Similar results were found when analysing the data according to male (paired t-test: mean of differences 0.982 ± 0.412; t(5) = 5.837, *p* = 0.0021) and female sex (Wilcoxon matched-pairs signed rank test: median of differences = 1.163 mm; W = 30.00; *p* = 0.0391). There were no statistically significant differences in the RMS values between male and female patients in their respective comparisons.

**Table 6:** Overview of the RMS of the condylar head between the various examinations.

Clinical parameter	Median; Mean ± SD	95% CI of mean	Comparison to T1–T2
RMS (in mm) for all patients			
T1–T2	1.84; 1.72 ± 0.51	1.49 to 1.95	
T2–T3	0.53; 0.72 ± 0.39	0.49 to 0.94	median of differences = 1.064 mm; W = 97.00; *p* = 0.0009^a^
T1–T3	1.70; 1.70 ± 0.55	1.39 to 2.02	median of differences = −0.031 mm; W = −25.00; *p* = 0.4631^a^
RMS (in mm) for male sex			
T1–T2	1.86; 1.90 ± 0.27	1.70 to 2.11	
T2–T3	1.03; 0.94 ± 0.46	0.46 to 1.42	mean of differences = 0.982 ± 0.412; t(5) = 5.837, *p* = 0.0021^b^
T1–T3	1.88; 1.84 ± 0.54	1.28 to 2.41	mean of differences = 0.080 ± 0.368; t(5) = 0.533, *p* = 0.6170^b^
RMS (in mm) for female sex			
T1–T2	1.71; 1.53 ± 0.61	1.12 to 1.94	
T2–T3	0.52; 0.55 ± 0.24	0.35 to 0.75	median of differences = 1.163 mm; W = 30.00; *p* = 0.0391^a^
T1–T3	1.66; 1.60 ± 0.57	1.12 to 2.07	median of differences = −0.109 mm; W = −20.00; *p* = 0.1953^a^
MAD (in mm) for all patients			
T1–T2	1.43; 1.38 ± 0.43	1.18 to 1.57	
T2–T3	0.45; 0.58 ± 0.34	0.39 to 0.78	median of differences = 0.892 mm; W = 95.00; *p* = 0.0012^a^
T1–T3	1.44; 1.38 ± 0.48	1.10 to 1.66	median of differences = −0.021 mm; W = −29.00; *p* = 0.3910^a^
MAD (in mm) for male sex			
T1–T2	1.43; 1.54 ± 0.27	1.34 to 1.75	
T2–T3	0.80; 0.77 ± 0.40	0.35 to 1.19	mean of differences = 0.808 ± 0.346; t(5) = 5.726, *p* = 0.0023^b^
T1–T3	1.58; 1.50 ± 0.49	0.99 to 2.02	mean of differences = 0.074 ± 0.321; t(5) = 0.564, *p* = 0.5969^b^
MAD (in mm) for female sex			
T1–T2	1.22; 1.22 ± 0.51	0.88 to 1.56	
T2–T3	0.40; 0.45 ± 0.23	0.26 to 0.63	median of differences = 0.819 mm; W = 30.00; *p* = 0.0391^a^
T1–T3	1.44; 1.29 ± 0.49	0.88 to 1.69	median of differences = −0.129 mm; W = −26.00; *p* = 0.0781^a^
MSD (in mm) for all patients			
T1–T2	−0.43; −0.34 ± 0.44	−0.54 to −0.14	
T2–T3	−0.14; −0.14 ± 0.26	−0.29 to 0.01	mean of differences = −0.134 ± 0.449; t(13) = 1.115, *p* = 0.2850^b^
T1–T3	−0.31; −0.28 ± 0.54	−0.60 to 0.03	mean of differences = 0.008 ± 0.372; t(13) = 0.084, *p* = 0.9346^b^
MSD (in mm) for male sex			
T1–T2	−0.51; −0.30 ± 0.44	−0.64 to 0.03	
T2–T3	0.00; 0.00 ± 0.27	−0.29 to 0.28	median of differences = −0.433 mm; W = −15.00; *p* = 0.1562^a^
T1–T3	−0.28; −0.14 ± 0.50	−0.67 to 0.39	median of differences = −0.134 mm; W = −17.00; *p* = 0.2188^a^
MSD (in mm) for female sex			
T1–T2	−0.43; −0.37 ± 0.48	−0.69 to −0.05	
T2–T3	−0.23; −0.25 ± 0.20	−0.42 to −0.08	mean of differences = 0.018 ± 0.412; t(7) = 0.121, *p* = 0.9073^b^
T1–T3	−0.45; −0.39 ± 0.58	−0.87 to 0.09	mean of differences = 0.162 ± 0.313; t(7) = 1.469, *p* = 0.1853^b^

a Wilcoxon matched-pairs singed rank test.

b Paired t test.

Comparable results could be confirmed for the MAD values. The MSD values showed no significant differences among the groups compared (Table 6).

### Miscellaneous

Throughout the observation period, no magnesium screws became exposed or protruded beyond the condylar surface.

Correlations between radiological and clinical variables were analysed using Spearman's rank correlation. There was a statistically significant positive association between age and absolute changes in volume (Spearman correlation: r(19) = 0.466, *p* = 0.038). However, multiple linear regression for age did not reveal any statistically significant association between the examined parameters. Additionally, multiple logistic regression for sex could not support any association either.

## Discussion

### Key points

Patients undergoing ORIF with HBS-Mg showed full dental occlusal restoration and significant improvements in interincisal distance and excursive movements over time. The fractured side treated with ORIF exhibited a shorter laterotrusion distance at all follow-ups except the first Cluster. Although statistically significant differences were observed, their clinical relevance in daily life was negligible.

The applied 3D examination method demonstrated good intraclass and interrater reliability. Significant changes in volume and surface area were observed in all patients. Sex-specific analyses were performed because sex is a recognised biological variable that may influence bone healing and remodelling. Given the limited sample size, these analyses were exploratory and did not identify statistically significant differences. Still, sex-based analysis revealed statistically significant changes in surface area only in female patients.

The quantitative three-dimensional analysis demonstrated RMS, MAD, and MSD surface deviations of 1.70 mm, 1.43 mm, and −0.43 mm between the immediate postoperative examination (T1) and the six-month follow-up scan (T2). The mean deviations between the six-month follow-up scan (T2) and the final, 12-month follow-up scan (T3) were 0.53 mm, 0.45 mm, and −0.14 mm, respectively. No differences in surface deviation were identified between the T1-T2 and T1-T3 scan comparisons, suggesting that the majority of condylar surface remodelling occurred within the first six months after ORIF and that the condylar surface remained stable one year postoperatively.

### Strengths and limitations

The strengths of this study include the combined evaluation of clinical and three-dimensional radiographic outcomes and the use of a reproducible segmentation and analysis workflow. Furthermore, all patients were treated using a standardised surgical approach and implant system.

Limitations include the retrospective design, the absence of a control group, the single-centre setting, and the relatively small sample size. In addition, radiographic follow-up was not available for all patients at all time points, which may have introduced selection bias.

Due to sample size constraints within the subgroups (9 men and 11 women), sex-specific analyses were treated solely as exploratory. Readers should approach these comparisons with caution, as the analysis was underpowered to detect moderate variances between sexes.

The study cohort comprised several AOCMF fracture subtypes, each with potentially distinct biomechanical characteristics and healing patterns. Owing to the limited sample size, meaningful subgroup analyses or adjustment for fracture subtype were not feasible. Consequently, fracture morphology may have contributed to the variability observed in postoperative remodelling and should be considered a potential source of residual confounding.

Only 60% of patients were available for the ≥360-day follow-up, introducing the possibility of attrition bias if patients lost to follow-up differed systematically from those who remained under observation.

Another limitation is that implant degradation itself was not directly quantified. Although serial three-dimensional imaging allowed assessment of postoperative condylar remodelling, the degradation behaviour of the magnesium screws was not analysed using dedicated measures such as implant volumetry, radiographic changes in implant morphology, corrosion products, or peri-implant gas formation. Consequently, the temporal relationship between implant degradation kinetics and condylar remodelling remains speculative. Future prospective studies combining quantitative implant degradation assessment with three-dimensional bone analysis may help clarify these relationships.

### Interpretation

Open reduction and internal fixation has increasingly become the preferred treatment for condylar head fractures, with recent systematic reviews and meta-analyses demonstrating superior functional outcomes compared with conservative management (8). Nevertheless, no consensus exists regarding the optimal fixation material (45). Owing to the complex anatomy of the condylar head, osteosynthesis devices in this region are subjected to considerable mechanical stress (1, 45).

Cannulated compression screws are an established method for stabilising small bony fragments to larger structures and have proven reliable and straightforward in trauma surgery (46, 47). They were first introduced for the treatment of CHFs in maxillofacial surgery in 2007 (48) and later applied to other mandibular regions, including the anterior body (49) and the mandibular angle (50). The use of titanium Herbert screws for condylar head fixation then largely disappeared from the literature until it was revisited more recently (35).

While some authors recommend routine removal of titanium fixation devices from the condylar head (51, 52), most suggest explantation only when loosening or unfavourable remodelling exposes the hardware (13, 20). Consequently, biodegradable fixation options have been introduced, including polylactic acid-based systems (23) and magnesium alloy implants (25, 26, 28, 29), rendering secondary implant removal surgery superfluous. Even long-term outcomes of osteosynthesis with biodegradable magnesium alloys have been reported, demonstrating excellent clinical results (30). The present study provides follow-up data up to one year for another biodegradable magnesium alloy system with a slender overall geometric profile and a modified PEO surface.

Magnesium is prone to corrosion, particularly in aqueous environments. Its degradation mainly occurs via anodic dissolution, producing magnesium ions (Mg^2^⁺), accompanied by cathodic hydrogen (H₂) release (53). Histological studies have shown that degradation begins within the first week after implantation, reaching approximately 50% by 12 weeks, during which moderate bone formation is observed around the implant. By 52 weeks, the metallic component is typically entirely degraded (54), making long-term follow-up beyond this period essential.

In the present cohort, radiographic evidence of peri-implant gas was detected in some patients in Clusters 3 and 4 but had resolved entirely by Cluster 5, consistent with reports from other groups (55, 56). Rather than being purely harmful, low levels of hydrogen release appear to promote periosteal cell proliferation, influence the local microenvironment, and support osseointegration. Accordingly, hydrogen has increasingly been proposed as a physiologically beneficial factor rather than merely a toxic by-product (57). However, these effects were observed in experimental studies, and their clinical relevance in fracture healing or other clinical settings remains to be established. Nonetheless, in the present study, none of the patients experienced adverse tissue reactions related to gas formation or reported subjective deterioration in well-being. Likewise, clinical assessments revealed no functional impairment. On the contrary, all patients established clear signs of satisfactory recovery of mandibular mobility, as discussed in the following section.

As expected, after ORIF of CHFs, a progressive increase in mouth opening and mandibular movements was observed. In particular, adequate interincisal distance is essential for effective mastication and consequently, sufficient nutritional intake. Reported values in the literature range from 37.8 ± 4.2 to 50.5 ± 5.1 mm (18, 21, 25, 30, 35, 51, 52), which is consistent with the measurements obtained in the present study.

Assessment of lateral and anterior mandibular movements after ORIF of CHF has been less frequently addressed. Nevertheless, available studies (18, 20, 25, 30, 35) and our own data indicate favourable outcomes across all planes of mandibular excursion. There were, however, significant differences in lateral excursion between the side treated by ORIF and the healthy, unaffected side in all but the first examinations. Even though the difference was present and significant, none of the patients complained of reduced mobility, difficulties with mastication, or impairment during their daily lives.

In addition to mandibular mobility, re-establishing normal occlusion after treatment of mandibular fractures is a second key prerequisite for adequate mastication and function. Previous studies have reported rates of occlusal disturbance after ORIF ranging from 0 to 10% (13, 21, 25, 30, 35, 51, 58). In our cohort, no persistent occlusal abnormalities were observed at final follow-up, consistent with published data.

Facial nerve dysfunction represents a serious complication of surgical management of CHFs. Reported rates of temporary and permanent palsy vary considerably in the literature (13, 18, 21, 30, 35, 58, 59). Importantly, long-lasting facial nerve deficits have most often been linked to secondary procedures for implant removal, likely due to scar formation and the increased difficulty of nerve dissection (60, 61). As in a previous study, biodegradable fixation was used, eliminating the need for explantation and likely contributing to the absence of persistent facial nerve palsy at final follow-up (30).

Three-dimensional assessment of the condylar head has been investigated since 2010 (62). Although several studies have attempted to quantify condylar head volume, absolute values are often not provided; instead, volumes are frequently reported relative to the total mandibular volume (63) or to the total ramus volume (64). Several investigations have specifically examined condylar head volume in the context of trauma or surgical management of the mandibular condyle and head (17, 35, 51, 52). Some of these studies describe only postoperative volume loss without absolute measurements (52), whereas others report absolute volumetric values at different follow-up time points (17, 30, 35, 51). In general, the absolute volumes reported in those studies are higher than those observed in the present cohort.

These discrepancies may partly be attributed to methodological differences, particularly variations in the segmentation planes used to define the condylar head (17, 51). Nevertheless, despite lower absolute volumes in our dataset, the relative volume changes were more pronounced than those reported previously, reaching 15.3% (17) and 15.9% (35). One possible explanation could be the use of biodegradable implants in this study, which may have elicited a biological response during degradation that does not occur with bioinert titanium. In fact, a study applying similar ORIF and imaging protocols reported comparable absolute volumetric changes, but observed volume increases rather than decreases. However, titanium screws were used in that investigation (35). Another study reported changes that were approximately on par with those presented in this study (30).

One study using biodegradable magnesium implants suggested that the implant volume, and thereby the amount of the inserted material, may influence changes in condylar head volume (30). The study presented here used smaller implants with a PEO-modified surface and still showed the same relative volume changes. Thus, the authors suggest that the amount of magnesium alloy introduced into the condylar head and the PEO-modified surface may not account for the extent of the observed volumetric changes. Introducing biodegradable implants may stimulate a biological response, which may account for the more pronounced changes in relative condylar head volume.

Only a limited number of studies have evaluated the condylar head surface area. One investigation analysed this parameter, but applied a more cranially located cut-off plane to define the condylar head than was used in the presented work (62). Another group assessed structural alterations of the condylar head after ORIF by measuring the distance between the fixation screws and the articular surface using three-dimensional datasets. However, this approach yielded only two-dimensional measurements in three planes and did not capture the true condylar surface, making direct comparison with our findings complex (52). In addition, studies that used similar imaging and segmentation protocols utilising titanium headless compression screws for CHFs reported lower changes in absolute and relative surface area values (35), which may again be attributable to differences in the biological inertness and degradability of the implants, as mentioned earlier. A study utilising magnesium alloy implants for CHFs reported similar changes in absolute and relative surface area of the condylar head (30). The authors believe that the reduction in the surface area of the condylar head serves as a surrogate marker of three-dimensional remodelling, indicated by a smoothening of the healing bone of the condylar head.

Analysis of 3D-congruence employing the RMS has previously been applied to assess mandibular ramus symmetry (64). More recently, it has been introduced as an objective method for quantifying three-dimensional changes after ORIF with titanium headless compression screws, whereby values derived from paired CBCT datasets reflect alterations in surface configuration rather than true volumetric plasticity, with reported differences of up to 1.6 mm (35). Another study identified the period with the most significant remodelling of the condylar head after ORIF with magnesium alloy headless compression screws to occur within the first 100 days – or approximately 3 months – after ORIF. RMS values ranged from 1.7 mm to 1.8 mm in the comparison of intervals (30). Another study confirms that the most pronounced remodelling of the condylar head occurs during the first 3 months following ORIF (65). Lastly, although a correlation between age and volumetric changes was observed, no independent predictors of radiological or clinical outcomes were identified. Compared with our previous investigations of both titanium and earlier magnesium-alloy fixation systems (30, 35), the present study provides important additional insight by evaluating a reduced-head, PEO-coated WE43 magnesium screw using the same quantitative three-dimensional assessment strategy. Contrary to our initial hypothesis, reducing implant volume and modifying the surface texture did not reduce postoperative condylar remodelling. This finding suggests that the extent of remodelling is unlikely to be determined solely by implant geometry or surface characteristics and may instead reflect the complex biological and biomechanical processes accompanying fracture healing. At the same time, the consistently favourable functional outcomes indicate that substantial three-dimensional remodelling can occur without clinically relevant impairment during the first postoperative year. These observations refine the current understanding of condylar healing following biodegradable magnesium fixation and provide an important basis for future implant optimisation.

Finally, the observed remodelling may, in part, be related to the gradual degradation of the magnesium implant and the associated local biological environment. However, the present study did not directly assess degradation kinetics or tissue-level biological responses, and, therefore, no causal relationship can be established. The proposed mechanism should be regarded as a biologically plausible interpretation based on previous experimental studies rather than a finding demonstrated by the present investigation.

## Conclusion

This retrospective observational cohort study suggests that fixation of CHFs with a reduced-head biodegradable WE43 magnesium screw with PEO surface modification is associated with favourable functional recovery and stable clinical outcomes at the one year follow-up. Significant three-dimensional remodelling of the condylar head was observed during follow-up but was not associated with clinically relevant functional impairment.

Given the absence of a control group and the limited sample size, direct comparisons with other fixation materials cannot be drawn. Nevertheless, the findings support the use of this implant design as a viable option for treating CHFs.

## Data Availability

The raw data supporting the conclusions of this article will be made available by the authors, without undue reservation.
